# Supplementary material for: Interplay between Mg2+ and Ca2+ at multiple sites of the ryanodine receptor
Source: Nat Commun. 2024 May 15;15:4115. doi: 10.1038/s41467-024-48292-3 (PMC11096358; doi:10.1038/s41467-024-48292-3)
Supplement: Supplementary file 1 — Supplementary Information [file 41467_2024_48292_MOESM1_ESM.pdf]

Supplementary Information for

Interplay between Mg^{2+} and Ca^{2+} at multiple sites of the ryanodine receptor

Ashok R. Nayak[1], Warin Rangubpit[2], Alex H. Will[1], Yifan Hu[1], Pablo Castro-Hartmann[1,5], Joshua J. Lobo[1], Kelly Dryden[3,6], Graham D. Lamb[4], Pornthep Sompornpisut[2], Montserrat Samsó[1*]

Affiliations
[1]Department of Physiology and Biophysics, Virginia Commonwealth University, Richmond, VA, USA.
[2]Department of Chemistry, Faculty of Science, Chulalongkorn University, Bangkok, Thailand.
[3]Department of Molecular Physiology and Biological Physics, University of Virginia, Charlottesville, Virginia, USA.
[4]Department of Microbiology, Anatomy, Physiology and Pharmacology, La Trobe University, Melbourne, Victoria, Australia.
[5]Present address: ThermoFisher Scientific, Cambridge, United Kingdom.
[6]Present address: Department of Chemistry and Biochemistry, UC Santa Barbara, Santa Barbara, CA, USA.
[*]Correspondence to: montserrat.samso@vcuhealth.org, pornthep.s@chula.ac.th

This PDF file includes:

Supplementary Figures 1 to 5
Supplementary Tables 1 to 3

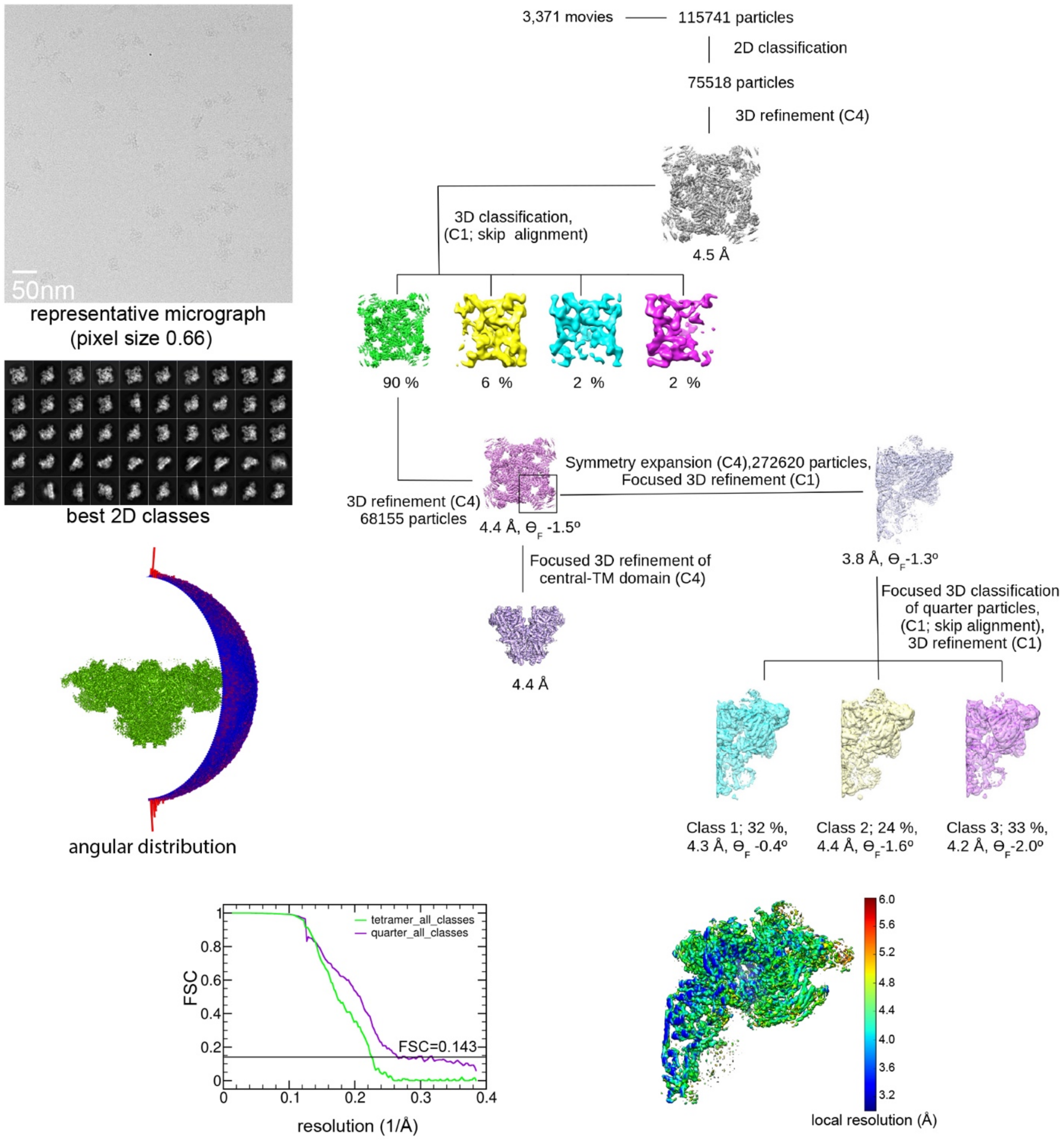

Supplementary Figure 1. Image processing scheme for the RyR1-ACP/LMg^{2+} dataset.

Flowchart depicting a representative micrograph collected on a Titan Krios at 105,000x magnification with a K2 camera in super-resolution mode, 2D class averages obtained by reference-free 2D classification, the entire RyR1 map, and maps focused on the central/transmembrane regions and quarter portion of RyR1. The Euler angular distribution of the particles contributing to the 4.4 Å map, the Gold standard Fourier shell correlation and local resolution of the symmetry-expanded map are also shown. Theta is the flexion angle.

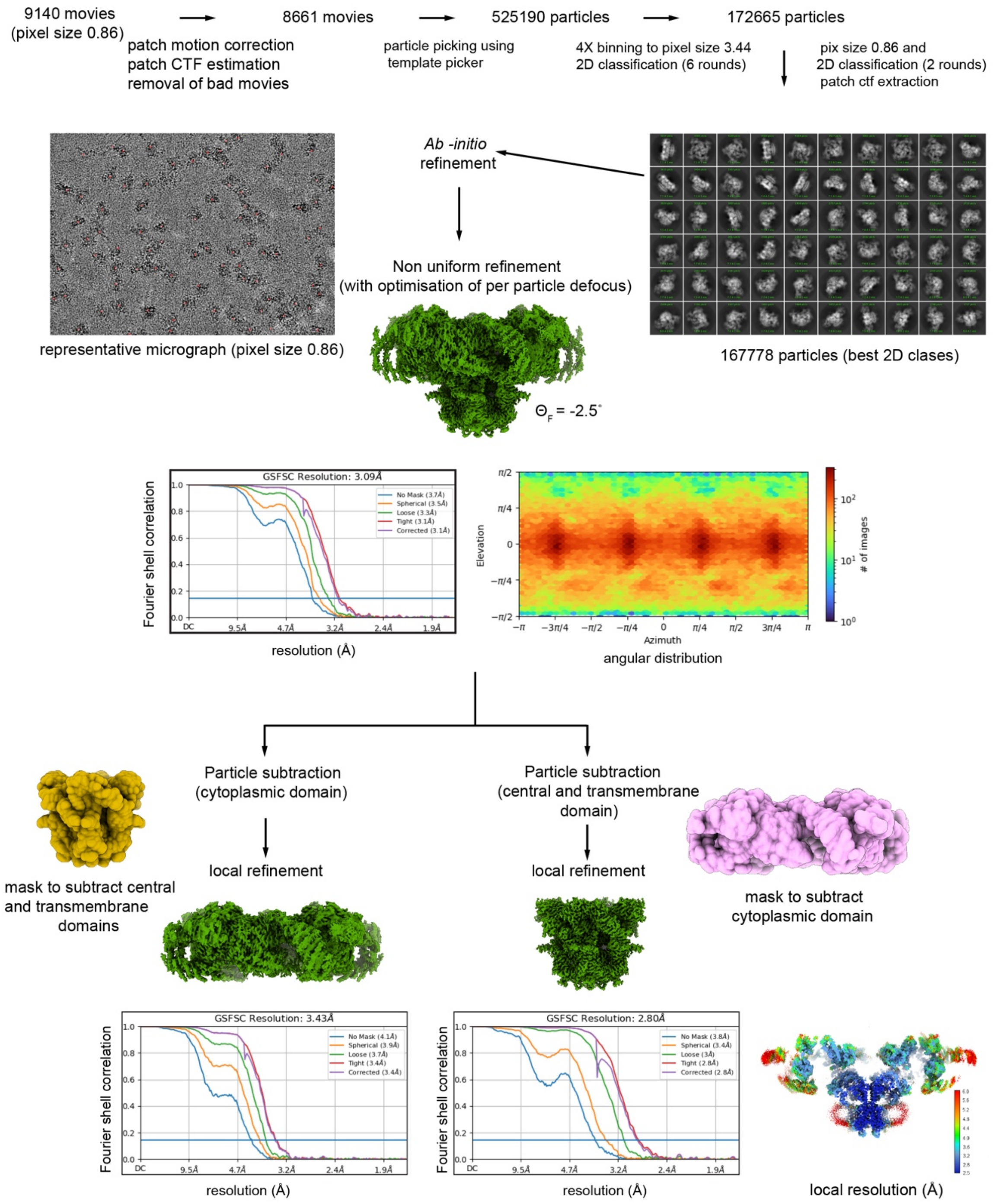

Supplementary Figure 2. Image processing scheme for the RyR1-ACP/HMg^{2+} dataset.

Flowchart depicting a representative micrograph collected on a Titan Krios at 105,000x magnification with a K3 camera in counting mode, 2D class averages after eight rounds of 2D classification, the entire RyR1 map, and maps focused on cytoplasmic and central/transmembrane regions obtained after particle subtraction. The masks used to generate high-resolution reconstructions via particle subtraction and local refinement are shown. The Gold standard FSC (GSFSC) curves of the corresponding cryo-EM reconstructions are depicted. Angular distribution and local resolution are also shown for the main map. Theta is the flexion angle.

MD replica number 2

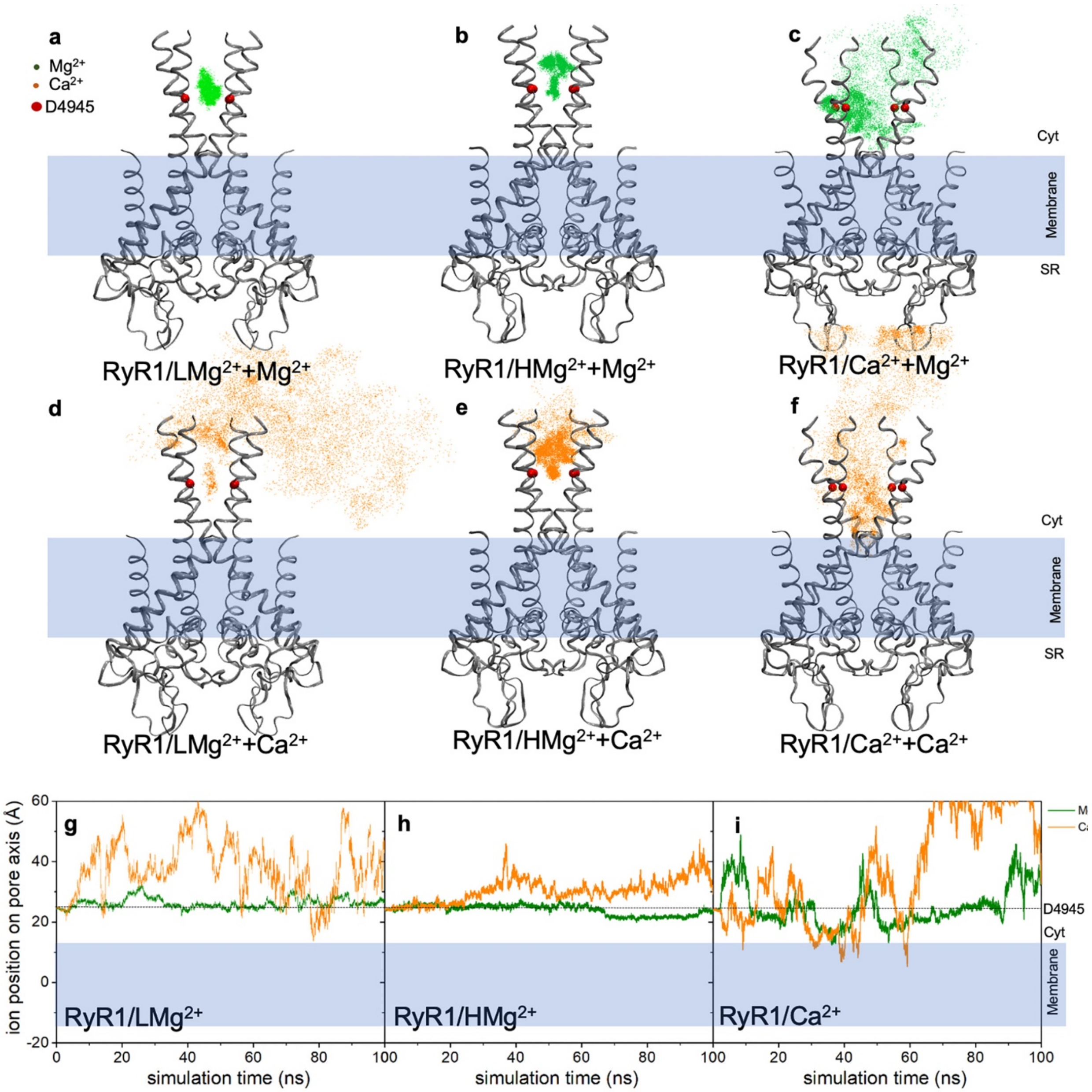

(see legend in the next page)

MD replica number 3

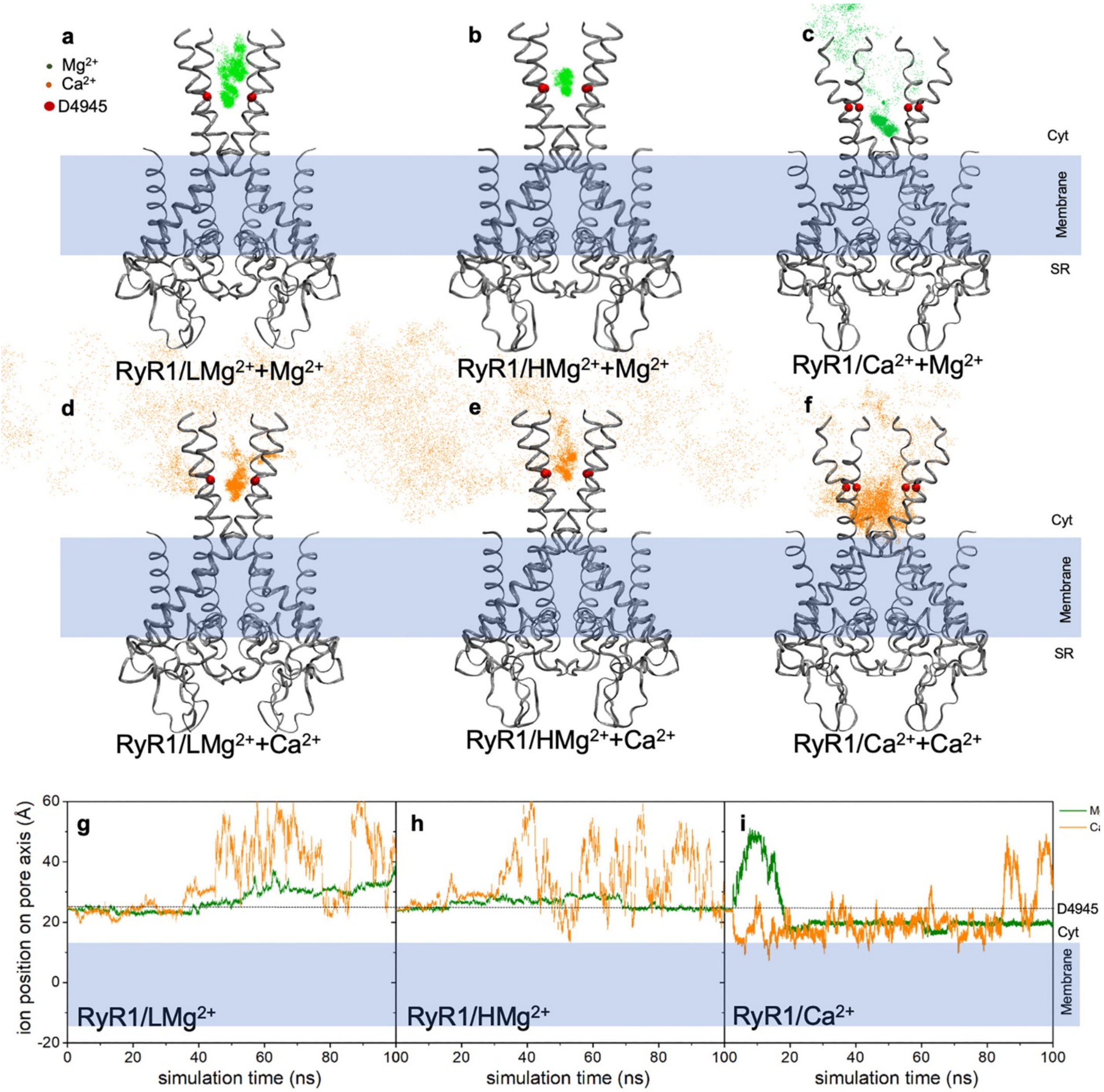

Supplementary Figure 3. Replicas of the molecular dynamics simulations of the pore domain.

Two replicas illustrating the reproducibility of the MD simulations. **a-f** Displacement of Mg^{2+} and Ca^{2+} ions is depicted with green and orange dots, respectively, at the D4945 site (red spheres) during 100ns MD simulations. Each dot represents the position of the indicated cation through the collection of MD snapshots taken at intervals of 0.02ns. The ribbon structures of the RyR1 pore domain in various conformations are illustrated in their initial configurations. **g-i** Ion displacement in relation to the z-axis of the channel over the course of simulation time. See also Fig. 4.

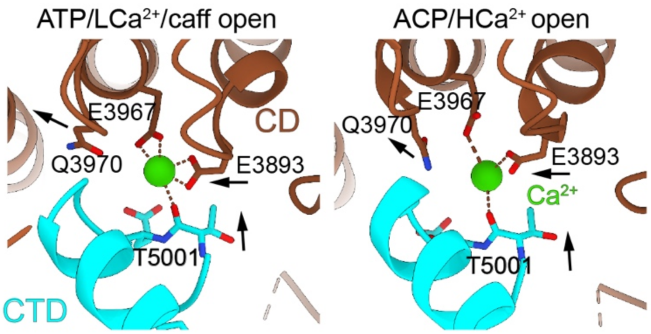

Supplementary Figure 4. Binding of Ca^{2+} at the high-affinity Ca^{2+} activation site under open-state conditions.

The high-affinity Ca^{2+} binding site at the CD/CTD interface of open RyR1 has similar configuration at low (30 μM) and high (2 mM) Ca^{2+} concentrations. The PDB IDs are 5TAL and 7TDH, respectively. Arrows indicate the Ca^{2+}-induced conformational change. Pore axis is on the left.

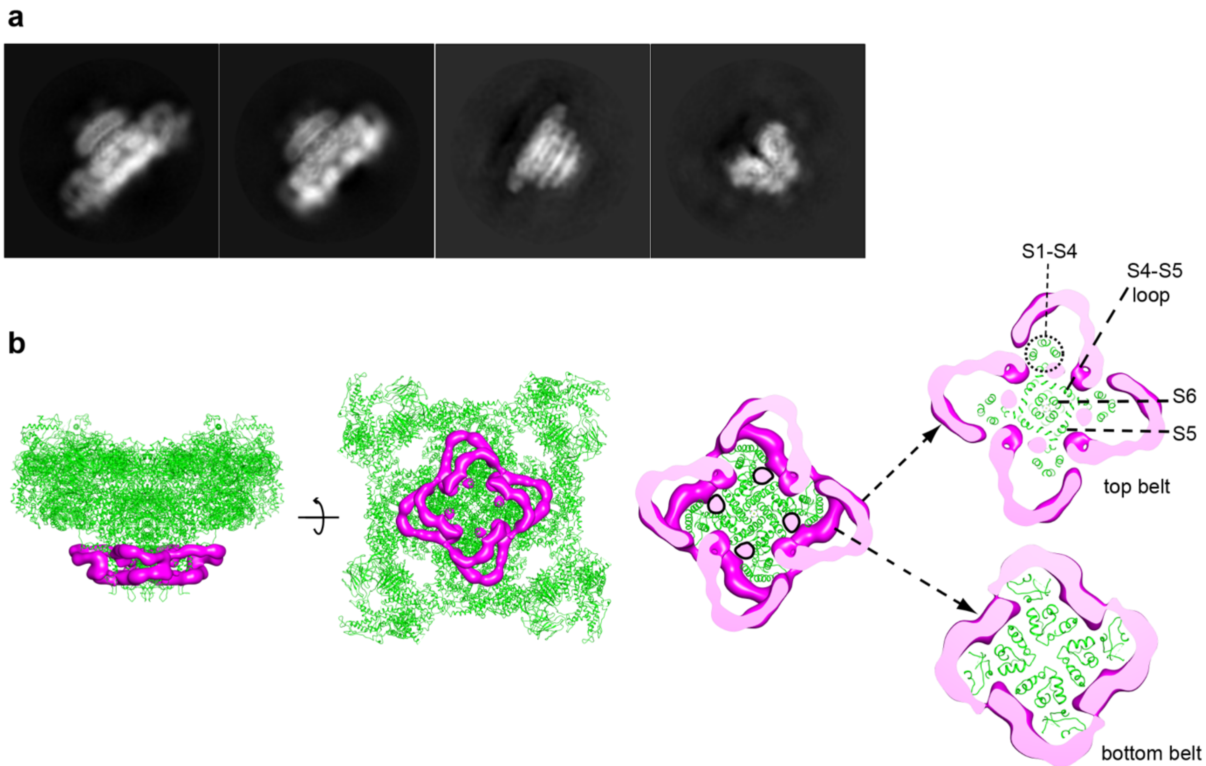

Supplementary Figure 5. Nanodisc and lipid densities surrounding the RyR1 TM domain.

a 2D class averages of the tetrameric map (top left) and CD-TMD focused map (top right) of RyR1-ACP/LMg^{2+} displaying the nanodisc region surrounding its transmembrane domain. The CD-TMD-focused reconstruction consists of the CD, EF, U-motif, TMD and CTD domains (residues 3668-5037). **b** Difference map corresponding to the nanodisc obtained by subtracting the model-derived simulated map from the cryo-EM focused map, superimposed to the RyR1-ACP/LMg^{2+} model (left, side view; center, luminal view; right, slices of the TMD at different levels of interest). The difference density attributed to the membrane scaffold protein (MSP1E3D1) consists of a top belt that surrounds each voltage sensor-like domain (S1-S4 bundle) plus lipids and a lower smaller belt following more closely the protein density with each segment surrounding one set of voltage sensor-like domains together with S5. Discrete non-protein densities, attributed to lipids, are shown in pink within the nanodisc.

Supplementary Table 1. Summary of cryo-EM data collection, image processing and model statistics.

	RyR1-ACP/LMg^{2+}	RyR1-ACP/HMg^{2+}
**Data Acquisition**		
Microscope/Detector	Krios/K2	Krios/ K3
Voltage (kV)	300	300
Magnification	105,000	105,000
Data collection mode	Super-resolution	Counting
Pixel Size (Å) (super-resolution)	1.32 (0.66)	0.86
Focus range (µm)	-1 to -2.5	-1 to -2
Total electron dose (e/Å^2) (Number of frames)	60 (60)	60 (40)
Total number of Micrographs	3,371	9,140
**Image Processing**		
Total number of Particles	115,741	525,190
Particles used (after 3D classification)	68,155	167,778
Resolution (Å) (symmetry expanded) [focused]	4.4 (3.8)	3.1 [2.8 TMD, 3.4 CytA]
Map sharpening B-factor (Å^2)	-250	-
EMDB ID	22615	26610
**Model Refinement**		
RMS Deviation (Bonds)	0.005	0.005
RMS Deviation (Angle)	0.95	1.125
Ramachandran Plot statistics (%)		
Preferred	91.99	93.43
Allowed	7.78	5.95
Outliers	0.23	0.62
**Model Validation**		
Clash-score	9.66	7.33
MolProbity Score	2.00	2.13
PDB ID	7K0S	7UMZ

Supplementary Table 2. Simulated systems for MD of the RyR1 Pore Domain[1] in the closed and open states with divalent cations

Model ID	PDB ID	State	Cations	Dimensions (Å^3)	Total atoms	Total molecules TIP3P/POPC
**1**	7K0S	RyR1/LMg^{2+} closed	Mg^{2+}	104×102×100	84846	17121/188
**2**	7UMZ	RyR1/HMg^{2+} closed	Mg^{2+}	104×102×100	84864	17171/187
**3**	7TDH	RyR1/Ca^{2+} open	Mg^{2+}	104×102×100	84478	17265/182
**4**	7K0S	RyR1/LMg^{2+} closed	Ca^{2+}	104×102×100	84807	17107/188
**5**	7UMZ	RyR1/HMg^{2+} closed	Ca^{2+}	104×102×100	84861	17169/187
**6**	7TDH	RyR1/Ca^{2+} open	Ca^{2+}	104×102×100	84466	17258/182

[1] The Pore Domain encompasses residues 4835-4956; see Methods section for further details.

Supplementary Table 3. DFT calculated binding energies of the $[M(H_2O)_n(D4945)_4]^{2-}$ complex of the RyR1 closed structures determined at high and low Mg^{2+} concentrations

System	State	$^1E_{AB}$ (Hartree)	E_A (Hartree)	E_B (Hartree)	ΔE_{bind} (Hartree)	ΔE_{bind} (kcal/mol)
**Before geometry optimization**						
$[Mg(H_2O)_6(D4945)_4]^{2-}$	HMg^{2+}	-2706.099	-658.527	-2047.455	-0.117	-73.56
$[Mg(H_2O)_6(D4945)_4]^{2-}$	LMg^{2+}	-2706.068	-658.517	-2047.450	-0.101	-63.22
$[Ca(H_2O)_7(D4945)_4]^{2-}$	HMg^{2+}	-3259.941	-1212.426	-2047.435	-0.080	-50.37
$[Ca(H_2O)_7(D4945)_4]^{2-}$	LMg^{2+}	-3259.941	-1212.462	-2047.419	-0.061	-38.13
**After geometry optimization[2]**						
$[Mg(H_2O)_6(D4945)_4]^{2-}$	HMg^{2+}	-2706.152	-658.482	-2047.519	-0.152	-95.10
$[Mg(H_2O)_6(D4945)_4]^{2-}$	LMg^{2+}	-2706.129	-658.473	-2047.509	-0.147	-92.13
$[Ca(H_2O)_7(D4945)_4]^{2-}$	HMg^{2+}	-3259.985	-1212.366	-2047.510	-0.109	-68.54
$[Ca(H_2O)_7(D4945)_4]^{2-}$	LMg^{2+}	-3259.985	-1212.389	-2047.512	-0.084	-52.73

[1]E_{AB}: energy of the $[Mg(H_2O)_6(D4945)_4]^{2-}$ or $[Ca(H_2O)_7(D4945)_4]^{2-}$, E_A and E_B : energies of $[Mg(H_2O)_6]^{2+}$ or $[Ca(H_2O)_7]^{2+}$ and $(D4945)_4^{4-}$, respectively. See Methods section for further details. For the conversion of the energy unit, 1 Hartree ≈ 627.5 kcal/mol.

[2]During DFT optimization, the structure tends to relax to a minimum energy state, which differs slightly from the structure obtained through MD. Notably, the optimized structure of $[Mg(H_2O)_6(D4945)_4]^{2-}$ in LMg^{2+} tends to show a geometry that converges towards that observed in HMg^{2+}.
