# Supplementary material for: Interplay between Mg2+ and Ca2+ at multiple sites of the ryanodine receptor
Source: Nat Commun. 2024 May 15;15:4115. doi: 10.1038/s41467-024-48292-3 (PMC11096358; doi:10.1038/s41467-024-48292-3)
Supplement: Supplementary file 3 — Description of Additional Supplementary Files [file 41467_2024_48292_MOESM3_ESM.pdf]

Description of Additional Supplementary Files

File Name: Supplementary Movie 1

Description: Effect of Mg^{2+} on the pore of RyR1 and tripartite hydrogen bond network.

File Name: Supplementary Movie 2

Description: MD simulation of RyR1/HMg^{2+} + Mg^{2+}: stabilization of Mg^{2+} by D4945 depicted in the cytoplasmic view.

File Name: Supplementary Movie 3

Description: MD simulation of RyR1/HMg^{2+} + Mg^{2+}: formation of hydrogen bond network among D4945, R4944, D4938, and Mg^{2+}, depicted in the side view.

File Name: Supplementary Movie 4

Description: Effect of Mg^{2+} and Ca^{2+} at the high affinity Ca^{2+} activation site and at the EF hand domain, and interrelationship between the EF hand domain and the S2-S3 loop (23L).
